# Circular RNA in cancer development and immune regulation

**DOI:** 10.1111/jcmm.16102

**Published:** 2020-12-05

**Authors:** Weizhen Li, Jia‐Qiang Liu, Ming Chen, Jiang Xu, Di Zhu

**Affiliations:** ^1^ Department of Laboratory Medicine Sixth Affiliated Hospital of Yangzhou University Taizhou China; ^2^ Department of Laboratory Medicine Affiliated Taixing Hospital of Bengbu Medical College Taizhou China; ^3^ Department of Oral and Cranio‐Maxillofacial Ninth People's Hospital School of Medicine Shanghai Jiao Tong University Shanghai China; ^4^ Department of Rehabilitation Huai'an Second People's Hospital The Affiliated Huai'an Hospital of Xuzhou Medical University Huai'an China; ^5^ School of Pharmacy and Shanghai Pudong Hospital Fudan University Shanghai China

**Keywords:** biomarker, cancer, circular RNAs, immunity, therapy

## Abstract

Circular RNAs (circRNAs) are a class of single‐stranded RNAs with closed loop structures formed by covalent bonds of head and tail. Exploration of circRNAs is continually increasing; however, their functional relevance largely remains to be elucidated. In general, they are stable, abundant, conserved and expressed in tissue‐specific manner. These distinct properties and their diverse cellular actions indicate that circRNAs modulate transcription and translation, and may even function as translation templates. Growing evidence reveals that circRNAs contribute to various physiological and pathological processes, including the initiation and progression of cancer. In this review, we present the current knowledge about circRNAs in cancer development, as well as their potential for use as biomarkers and even therapeutic targets. CircRNA’s role in immune regulation and antitumour immunotherapy is also discussed. In addition, possible challenges in antitumour therapy are raised, and current progress and future perspectives are provided.

## 1 INTRODUCTION TO CIRCULAR RNA

Circular RNA (circRNA) has received increased attention in recent times, although it was first found in the 1970s and initially considered to be an abnormal and functionless RNA splicing product.^1^, ^2^ CircRNA belongs to the non‐coding RNA family and has a closed loop structure formed by covalent bonds of head and tail. Lacking 3’ termini makes it insusceptible to exonuclease digestion, and thus more stable than associated linear mRNA.^3^ The development of technology and bioinformatics has facilitated the discovery of numerous circRNAs of different lengths and types, and research indicates that they are enriched in eukaryotic cells and highly conserved across species.^4^

Both circular RNAs and linear RNAs are derived from precursor mRNAs and transcribed with the same efficiency.^5^ Unlike linear RNAs’ classical splicing, circRNAs are generated by different modes, primarily by back‐splicing. According to the location of splice junction in genome, circRNAs are classified into four basic types, including exonic, intronic, exonic‐intronic and tRNA intronic. Exonic circRNAs (ecircRNAs) are formed by either single or several exons and account for the main body of cirRNAs; circular intronic RNAs (ciRNAs) are made up of introns alone; exonic‐intronic circRNAs (EIciRNAs) are composed of exons and introns; and tRNA intronic circRNAs (tricRNAs) are derived from splicing of pre‐tRNA introns.^5^, ^6^ Different types of circRNAs are distributed in different sites of cells. Generally, exonic circRNAs are located in cytoplasm, whereas some ciRNAs and EIciRNAs are in the nucleus, which is consistent with the location of their different biological functions, as shown below. Furthermore, circRNAs are reported to be packaged and released in certain vesicles (exosomes and microvesicles), and compared with in cells, circRNAs in exosomes are more enriched and widely expressed.^7^, ^8^

**Figure 1:**
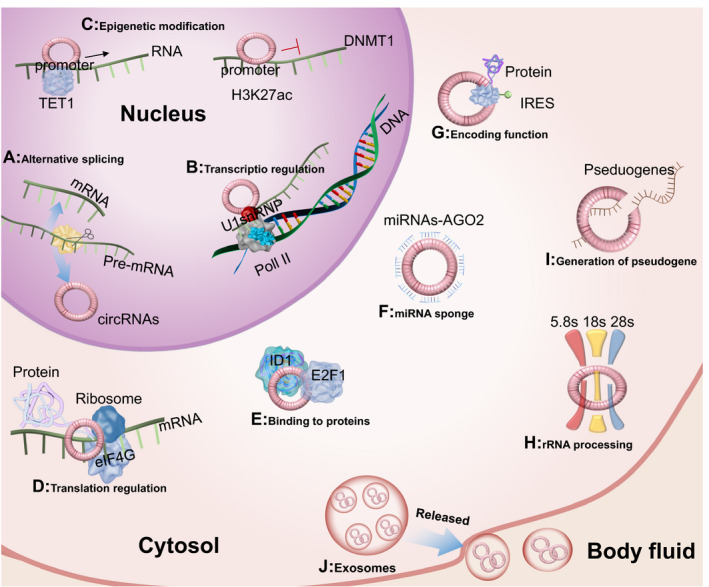
Illustration of Circular RNA function. Circular RNAs fulfil multiple functions. In nucleus, circRNAs can regulate transcription A, compete with the mRNA for the available splicing machinery and interfere in the alternative splicing process; B, regulate the transcription of their gene of origin through direct (circRNAs) or U1 snRNP‐mediated (EIcircRNA) interaction with the RNA polymerase II; C, regulate parental gene by epigenetic mechanism. In cytoplasm, circRNAs can D, regulate the translation of mRNAs by interacting with some translation initiation associated proteins; E, bind to some proteins as protein sponge or scaffold to affect their functions or translocations; F, act as miRNAs sponges, a majority of circRNAs function that interact with miRNA‐AGO2 complexes to affect miRNA functions; G, encode proteins or peptides, possibly with an internal ribosome entry site (IRES) or with m^6^A modification; H, interfere with the processing of pre‐rRNA subunits; I, generate pseudogenes. J, circRNAs may be released for cells communication or acting as vectors for miRNAs or proteins

Although most of the biological functions of circRNAs have yet to be elucidated, the current knowledge about circRNAs has shown that they can regulate gene expression (transcription and translation), interact with proteins, act as miRNA sponges, translate proteins or peptides, be involved in rRNA processing and generate pseudogenes (Figure 1). In the nucleus, circRNAs function in the following: (A) competition in splicing. When the circRNA contains the same exon as the parental gene, circRNAs can compete with linear splicing of pre‐mRNA, thereby affecting the level of linear RNAs.^5^ (B) Transcription regulation: ciRNAs and EIciRNAs can bind to U1 snRNP through RNA‐RNA interactions and further interact with the Pol II transcription complex to enhance parental gene expression.^9^ (C) Regulation of parental gene by epigenetic mechanism: for example, circFECR1 from the FLI1 gene can bind to FLI1 promoter, recruit TET1 demethylase and bring extensive DNA demethylation in the CpG islands of promoter to activate FLI1. FECR1 can also directly inhibit the gene transcription of DNMT1 methyltransferase, an essential enzyme in DNA methylation maintenance, by binding to its promoter region, which is enriched in H3K27ac.^10^ In cytoplasm, circRNAs function in (D) translation regulation: for example, CircYap can bind with Yap mRNA and the proteins associated with translation initiation, eIF4G and PABP, thereby suppressing Yap translation initiation.^11^ (E) Binding to proteins: some circRNAs that have binding sites for RNA‐binding proteins may serve as protein sponges or scaffolds to affect their functions or translocations. For example, circ‐Foxo3 can bind both Foxo3, p53 and sponge for MDM2. This allows it to prevent Foxo3 ubiquitylation or to act as scaffold to facilitate MDM2‐dependent p53 ubiquitylation.^12^ In other cases, circ‐Foxo3 can bind the transcription regulators ID1, E2F1, FAK and HIF1α, and prevent their nuclear translocation.^13^ In this function, circ‐Foxo3 does not affect the expression level of these transcription factors, but affects their localization. (F) Acting as miRNA sponges or decoys: highly abundant circRNAs contain diverse miRNA‐binding sites, and whether the effect of sponging inhibits or protects the target miRNA may be dependent on the cellular context,^14^ which is true for the majority of circRNA functions. (G) Templates for translation: circRNA was initially regarded as a class of endogenous non‐coding RNA that could not translate proteins, and it was considered to lack essential elements for cap‐dependent translation. Recently, several circRNAs have been shown to encode proteins or peptides in other cap‐independent manners, such as circMbl, circFBXW7 and circZNF609.^15^, ^16^ It may translate either from an artificial internal ribosomal entry site (IRES) within an open reading frame (ORF), or following the incorporation of m6A RNA modification in the 5′ untranslated region (UTR).^17^ (H) Involvement in rRNA processing: for example, circANRIL can bind to pescadillo homologue 1 (PES1), an essential 60S‐preribosomal assembly factor, resulting in the impairment of exonuclease‐mediated pre‐rRNA processing and ribosome biogenesis. (J) CircRNAs can also generate pseudogenes like linear RNAs, such as circRFWD2‐derived pseudogenes.^18^ In addition, circRNAs in exosomes can also be released for cell communication or can act as vectors for miRNAs or proteins, functions which are described below.^19^

Based on these biological functions, circRNAs contribute to diverse physiological and pathological processes, notably in the onset and progression of cancer.^20^, ^21^ In this review, we discuss the crucial roles of circRNAs pertaining to cancer, including their potential value as biomarkers and therapeutic targets. In view of the involvement of immune system in cancer, we also review the role of circRNAs in immune regulation and antitumour immunotherapy.

## 2 CIRCULAR RNA IN CANCER DEVELOPMENT

**Figure 2:**
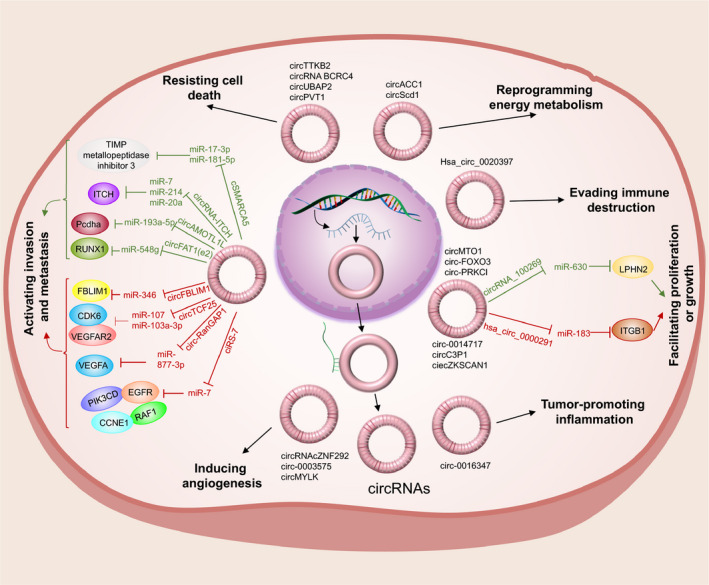
Illustration of Circular RNA in cancer. CircRNAs have been indicated (mainly in the cytoplasm) to contribute to various aspects of cancer progression. For example, some circRNAs may be involved in proliferation, growth, invasion, and metastasis. Tumour suppressor circRNAs are indicated by green lines and tumour‐ promotor circRNAs are shown by red lines

As a complex pathological process, cancer development contains many hallmarks, such as genome instability and mutation, reprogramming energy metabolism, sustaining proliferative signalling, enabling replicative immortality, evading growth suppressors, resisting cell death, activating invasion and metastasis, inducing angiogenesis, promoting tumour inflammation and evading immune destruction. Findings suggest that circRNAs are involved in these aspects of cancer development^12^, ^22^, ^23^, ^24^, ^25^, ^26^ (Figure 2).

### 2.1 Tissue specificity

Research into the relationship between circRNAs and cancer found that circRNAs are often dysregulated in cancers which allows tumour tissue to be distinguished from adjacent normal tissue.^20^, ^27^ Studies have identified multiple functional cancer‐associated circRNAs, with differential expression, in different cancers. These circRNAs act as tumour suppressors or promotors and impact cancer phenotypes in diverse ways. For example, ciRS‐7, acting as an oncogene, was found to be up‐regulated in colorectal cancer (CRC) tissues, whereas overexpression of ciRS‐7 in vitro could induce a malignant phenotype.^14^ Conversely, circ‐ITCH, as a sponge of oncogenic miR‐7 and miR‐214, was reported down‐regulated in lung cancer. Also, circ‐ITCH overexpression could suppress lung cancer cell proliferation.^28^ In addition, circRNAs are ubiquitous across different species, tissues and cell types, and have a tissue‐ and developmental stage‐specific expression pattern,^21^, ^29^ which may depend on the specific function of circRNA itself in its context. Xia et al^21^ first performed a global view of tissue‐specific (TS) circRNAs. They determined the TS circRNAs in human and mouse, identified their genomic location, conservation and binding of miRNAs or proteins to understand their function. Finally, they established an integrated database “TSCD” (Tissue‐Specific CircRNA Database: http://gb.whu.edu.cn/TSCD) as a better platform for characterizing features of TS circRNAs.

### 2.2 Invasion and metastasis

Invasion and metastasis play central roles in the progression of cancer. Indeed, a variety of circRNAs contribute to this process.^28^, ^30^, ^31^, ^32^, ^33^, ^34^ Zhong et al^27^ investigated the up‐regulated circTCF25 in bladder cancer (BC), and they found that circTCF25 overexpression could promote proliferation and migration both in vitro and in vivo through sequestering miR‐103a‐3p and miR‐107, and thus increasing CDK6 expression. Lu et al^35^ studied the up‐regulation of circ‐RanGAP1 in gastric cancer (GC). The loss‐ or gain‐of‐function experiments indicated that circ‐RanGAP1 was closely related to GC cell invasion and migration by sponging miR‐877‐3p to up‐regulate vascular endothelial growth factor A(VEGFA) expression. Furthermore, they found more circ‐RanGAP1 in plasma exosomes, and these exosomes with cargo could enhance GC cell migration and invasion capabilities, which suggested that exosomes can maintain the properties and original functions of circRNA, even transporting it as vectors. As mentioned above, the new circular RNA FECR1was identified when Chen et al^10^ explored the mechanism of Friend leukaemia virus integration 1 (FLI1), which was up‐regulated and acted as a tumour metastasis driver in breast cancer. They found circFECR1 in the FLI1 promoter chromatin complex, and this circular RNA could enhance metastasis in breast cancer by epigenetic mechanisms rather than canonical oncoprotein pathway. As shown in Figure 2, a variety of circRNAs contribute to metastasis and invasion processing through diverse mechanisms via interacting with different molecules.

### 2.3 Angiogenesis

Tumour progression usually requires formation of new blood vessels to supply nutrients and dispose of waste. CircRNAs are implicated in diverse tumorigeneses including angiogenesis. Zhong et al^36^ reported the up‐regulation of circRNA‐MYLK in BC and found that it could function as sponge of miR‐29a and activate target VEGFA. Ectopically overexpressing circRNA‐MYLK could facilitate cell proliferation, migration and tube formation of human umbilical vein endothelial cells (HUVEC) in vitro and promote tumour growth, metastasis and angiogenesis of BC xenografts in vivo. Li et al^37^ discovered that the up‐regulation of Hsa_circ_0003575 in impaired vascular endothelial cells was caused by oxidized‐low density lipoprotein (oxLDL), whereas Hsa_circ_0003575 silencing could enhance proliferation and angiogenesis capacity of HUVES. Moreover, hypoxia is believed to be a notable stimulus for angiogenesis^38^; thus some studies focused on the influence of hypoxia on endothelial cells and circRNAs in hypoxic cancer cells. Boeckel et al^23^ identified several circRNAs in hypoxic human endothelial cells, and circZNF292 especially exhibited proangiogenic activities in vitro, whereas circZNF292 silencing led to reduced tube formation and spheroid sprouting of endothelial cells. These are also consistent with the finding that tumour cells and vascular endothelial cells can, respectively, secrete relevant factors to promote each other's growth.^39^

### 2.4 Genome mutation

In general, accumulation of mutations accompanies the vast major of cancers. With an increase in studies focused on circRNAs in cancer, some circRNAs have been found to be associated with these mutations and post‐translational modifications. Mut‐p53 is known as the most common mutation in human cancers. Verduci et al^40^ showed that, as an oncogene, circPVT1 was up‐regulated in head and neck squamous cell carcinoma (HNSCC) and regulated by YAP through forming the YAP/circPVT1 complex. However, this complex requires mut‐p53 to function as the stabilizer. In addition, genetic alterations can also directly impact non‐coding RNAs including producing aberrant circRNAs. When the back‐splicing of circRNAs is upon chromosomal translocation, it will result in generation of fusion circRNAs (f‐circRNA). Guarerio et al and Tan et al,^41^, ^42^ respectively, found F‐circM9, F‐circPR and F‐circEA/F‐circEA‐2 in leukaemia and solid cancer. Additionally, functional analysis showed that they also contributed to oncogenesis. Recently, Liu et al^43^ systematically characterized circRNAs in the human dorsolateral prefrontal cortex and analysed circRNA expression variation by detecting partial quantitative trait loci (circQTLs) SNPs. Some circQTL SNPs exhibited an effect on circRNA formation by changing the canonical splicing site and further phenotypic changes. A similar approach is proposed to apply in cancer to identify whether these circQTL SNPs are involved in diverse tumorigenesis. In brief, some circRNAs may be involved in cancer genome mutations either through interacting with the mutant products, or via genetic alteration itself.

## 3 CIRCULAR RNA IN CANCER IMMUNE REGULATION

The immune system is responsible for defending the host from exogenous invasion and maintaining internal homeostasis. The emerging research on circRNAs has suggested that they are involved in the modulation of different immunocytes and diverse immune responses. For example, circular RNA100783 functions in CD8(+) T cell ageing and immunosenescence, and circRNA‐003780 and circRNA‐010056 function in macrophage differentiation and polarization.^44^, ^45^ A gene ontology analysis for 422 circRNAs identified from healthy human saliva categorized them into several groups, including establishment of T cell polarity, chemotaxis, inflammatory response and the integrin‐mediated signalling pathway, which indicated that these circRNAs may participate in intercellular signalling and immune responses.^22^ However, inflammation can play vital roles during all stages of tumour development, especially in early tumour initiation as well as tumour promotion, tumour progression and metastasis. Immunosuppressive cells, inflammatory cytokines and signalling, chemokines and inhibitory receptors all contribute to tumour‐promoting inflammation.^46^

### 3.1 Checkpoint

Immune checkpoints, such as PD‐1 and CTLA‐4 (inhibitory receptors), account for tumour immune escape. The rising hotspot of checkpoint blockade therapy and the emerging role of circRNAs has attracted studies seeking to elucidate the relationship between them. For instance, hsa_circ_0020397 was found to be up‐regulated in CRC and could mediate colorectal cancer cell viability and invasion by inhibiting miR‐138 and subsequently enhancing telomerase reverse transcriptase (TERT) and PD‐L1.^26^ In another case, circ‐UBAP2 was found to be differentially expressed in pancreatic adenocarcinoma (PAAD) and could modulate the expression of CXCR4 and ZEB1, which are linked with M2 macrophages, exhausted T cells and T‐regulatory cells (Tregs), and are positively correlated with PD‐1 and CTLA‐4.^47^ This relationship provides another perspective to apply PD‐1 in immunotherapy.

### 3.2 Chemokine

Chemokines are a type of cytokine that can recruit leucocytes and other types of cells. Evidence shows that some chemokines and chemokine receptors are frequently involved in cancer, even as tumour promoters. In one experiment, two human CRC cell lines were co‐stimulated by chemokines CCL20 and CXCL8, which resulted in CRC cell migration, invasion and the epithelial‐mesenchymal transition (EMT) process, accompanied by down‐regulation of circ_0026344.^48^ In another case, Zhang et al^49^ found that circFGFR1 could positively regulate CXCR4 expression in NSCLC cells. Knockout and enhancement experiments suggested that controlling the CXCR4‐related pathway in NSCLC cells might facilitate anti‐PD‐1 immunotherapy. Zhang et al^50^ explored the relationship between circ_0067934 and CXCR1 in thyroid cancer (TC). The results indicated that circ_0067934 down‐regulation expedited cell apoptosis and repressed cell proliferation, migration and invasion by inhibiting miR‐1304 and modulating CXCR1 expression.

### 3.3 Inflammatory cytokines

Cytokines in tumour development are typically derived from cancer cells or immunosuppressive cells. In the process of tumour inflammatory microenvironment formation, caspase‐1 contributes to the activation of proinflammatory cytokines IL‐1β and IL‐18, which induce the key downstream inflammatory response.^51^ Jin et al^52^ reported that circ‐0016347 served as a tumour promoter in osteosarcoma cells by sponging miR‐214 and enhancing the downstream target caspase‐1, followed by enhancing the inflammation‐associated mechanism. In another case, Liu et al^53^ found that overexpressed hsa_circRNA_002178 could sponge miR‐328‐3p and thus up‐regulate COL1A1, which resulted in progression of breast cancer, whereas hsa_circRNA_002178 silencing could inhibit tumour inflammation by decreasing the levels of IL‐6 and TNF‐α, preventing tumour growth.

### 3.4 Anti‐viral

A number of viruses have been proven to generate circRNAs after infecting the host, and some oncogenic viruses can encode circRNAs, which may contribute to oncogenesis, such as circEBNA_W1_C1 derived from Epstein‐Barr virus (EBV) and circE7 derived from human papillomavirus (HPV).^54^, ^55^ Under normal conditions, innate immunity provides the first defence against viral infection. Some dsRNA‐binding proteins, such as retinoic acid‐inducible gene‐I (RIG‐I), can recognize specific viral RNAs by their 5ʹ‐ppp and double‐stranded structure, and can also identify the exogenous circRNAs as nonself circRNAs. Additionally, the endogenous or host circRNAs are combined with RBPs, such as NF90/110, during biosynthesis in the nucleus, which enables self‐circRNAs to evade recognition and be distinguished from exogenous circRNAs. Takanobu et al identified differentially expressed host circRNAs in cells infected by Kaposi's sarcoma herpesvirus (KSHV), and they found that hsa_circ_0001400 activated by infection could inhibit expression of key viral genes LANA and RTA, and thus facilitate anti‐viral immune response.^56^ Li et al revealed that the host circ‐RNP may resist viral infection, whereas the viral infection could suppress its biogenesis through export of NF90/NF110 from the nucleus. There may be a competitive relationship between host circRNAs and viral infection.^57^, ^58^ Furthermore, based on host‐virus interaction, Ghosal et al^59^ constructed an integrated database, “HumanViCe” (http://gyanxet‐beta.com/humanvice), which could support ceRNA networks in virus‐infected cells, including those circRNAs enriched in pathways related to host immune responses and viral entry and replication.

## 4 CIRCULAR RNA AS A CANCER BIOMARKER

Considering the harmful effects of cancer, finding new and effective biomarkers to utilize in diagnostics, prognosis and/or to predict treatment response is vitally important. As described above, circRNAs are closely linked with cancer onset and progression, and there are differential expressions between tumour tissues and adjacent normal tissues. Furthermore, circRNAs have distinct properties of abundance, stability and tissue‐specific expression. More importantly, circRNAs can be secreted in human body fluids, such as saliva, urine, blood and cerebrospinal fluids, and are enriched in exosomes. Taken together, these characteristics indicate the advantages of circRNAs as biomarkers for detection and surveillance in cancer, which may be more accurate, convenient and noninvasive.^3^, ^4^, ^60^

### 4.1 Diagnostics

A number of tumour‐associated circRNAs have been researched to examine their diagnostic value in cancer. For example, circPVT1 was found significantly up‐regulated in osteosarcoma (OS) tissues, serum and chemo‐resistant cell lines. Also, the receiver operating characteristic (ROC) curve illustrated that it had advanced diagnostic value with greater specificity and sensitivity than alkaline phosphatase (ALP) in OS.^61^ Li et al^62^ systematically performed a literature search, which demonstrated that plasma circRNAs have higher diagnostic accuracy than tissue and that combined circRNAs have better diagnostic efficacy than single circRNAs. The utility of circRNAs for diagnostic procedures is currently being evaluated in a clinical trial (NCT03334708), which compares the diagnostic value of a panel of circRNAs and other molecules(proteins and proteases, circulating tumour DNA, exosomes, functional DNA repair assays, stromal elements) for early‐stage pancreatic cancer.^63^

### 4.2 Prognosis

Many groups have also investigated the prognostic value of circRNAs in cancer. Okholm et al^64^ identified 113 differentially expressed circRNAs between low‐ and high‐risk groups of patients with non‐muscle‐invasive bladder cancer (NMIBC). They also discovered 13 circRNAs that independently correlated with BC progression, especially circCDYL and circHIPK3, which exhibited high prognostic value for early‐stage BC. Exosomes can be perfect carriers for circRNAs and exo‐circRNAs derived from tumours and can be secreted into human body fluids, ultimately impacting cancer development.^8^ Li et al^65^ found that exo‐circ‐PDE8A derived from the blood of patients with pancreatic cancer was significantly up‐regulated, and these blood exosomes extracted from pancreatic ductal adenocarcinoma (PDAC) patients exhibited high correlation with duodenal invasion, vascular invasion, TNM stage, and finally, survival expectancy. In this way, exo‐circPDE8A may be a superior early diagnostic and prognostic biomarker for PDAC.

In addition, circRNAs also exhibit certain potential in predicting treatment response. Recently, Liu et al^66^ identified 1377 differentially expressed circRNAs between gefitinib‐effective and ineffective groups of non‐small‐cell lung carcinoma (NSCLC) patients. Among them, up‐regulated hsa_circ_0109320 was clearly correlated with longer progression‐free survival in gefitinib‐treated NSCLC patients. This provides a new approach to guide the clinical use of gefitinib. Moreover, studies into circRNAs and drug resistance also have indicated that circRNAs are implicated in the process of drug resistance in cancers by mediating diverse regulatory pathways and processes. For example, circPVT1 exhibits up‐regulation in patients with gastric cancer and cisplatin resistance during treatment, representing its potential as a biomarker related to cisplatin resistance. Circ_0081001 presents with gradual up‐regulation in patients with OS during treatment with cisplatin, indicating its potential in dynamic monitoring.^67^ The sensitivity and specificity of circRNAs for monitoring drug resistance should be taken into account.

## 5 CIRCULAR RNA IN CANCER THERAPY

Exploring novel antitumour methods is urgent due to the harm and severity of cancer and the limitations of traditional treatment methods. CircRNAs are widely involved in tumorigenesis, making them an attractive target in the field of cancer therapy.

### 5.1 Gene therapy

There have been multiple circRNAs identified as tumour promotors or suppressors, which influence cancer phenotypes in different ways, especially angiogenesis, proliferation or growth, invasion and metastasis. Exogenous up‐regulation or down‐regulation of relevant circRNAs, by changing their gene expression, is common in studying their potential in ameliorating harmful phenotypes. In contrast, plasmid or lentiviral vectors are often used to increase circRNAs levels. For example, circ‐Foxo3 was reported to be down‐regulated in bladder cancer, whereas overexpression of circ‐Foxo3 by the above vectors could induce bladder cancer apoptosis through directly inhibiting miR‐191.^68^ Similarly, as a translation template of FBXW7‐185aa, the circ‐FBXW7 level was found to be reduced in glioblastoma, whereas overexpression of FBXW7‐185aa could help inhibit cancer cell proliferation.^16^ However, RNA‐mediated interference (RNAi) and antisense oligonucleotides (ASO) are used to decrease circRNA levels, and the CRISPR/Cas9 system can even directly knockout the specific circRNAs. Cao et al^69^ reported the elevation of hsa_circ_0000291 in GC cell lines and found that silencing hsa_circ_0000291 could target the miR‐183/ITGB1 axis, resulting in inhibition of GC cell proliferation and metastasis. Yang et al^70^ identified a novel function of circ‐Amotl1, which facilitates c‐myc nuclear translocation through binding protein and regulates its gene transcription. Silencing circ‐Amotl1 could decrease cancer cell proliferation and increase cell apoptosis. Additionally, based on the knowledge of m^6^A modification in the translation of circZNF609, Hung et al recently found that m^6^A‐generating enzyme METTL3 knockout or deleting one Alu element to completely abolish circRNA splicing did not influence translation or detectable protein products, suggesting the existence of a METTL3‐independent initiation mechanism. Alternatively, other m^6^A‐generating enzymes could exist, and circRNA overexpression constructs may undergo *trans*‐splicing. Therefore, such tools need to be evaluated carefully, and further research may help us better apply gene therapy with this strategy.^83^

### 5.2 Therapeutics vectors

In addition to being targets in gene therapy, circRNAs can also be used as promising therapeutic vectors due to their unique stability and capacity for binding miRNA or proteins. They can be designed with miRNAs and/or proteins binding sites, which might be a simple, effective and convenient treatment strategy. Liu et al^71^ were the first to successfully synthesize a circular RNA, scRNA21, which had the specific binding site of miR‐21. After transfection of scRNA21, three types of GC cells all increased apoptosis. In this way, synthetic circRNA targeting multiple miRNAs, proteins or the combination of the two may impair multiple oncogenic pathways. Thus, their group furtherly constructed a novel circRNA sponging both miR‐21 and miR‐93, which led to significant inhibition of cancer cell proliferation and migration in vitro and remarkable suppression of tumour growth in vivo.^72^ Here, synthetic circRNA can be delivered by tumour cell exosomes as an agent, which can improve their targeting efficiency and increase their delivering amount. Furthermore, as transcription of circRNAs commences at Pol II promoters, another solution worth considering is designs with cell‐specific promoters or disease‐activated control elements, which may make the circRNAs optionally expressed in certain cells. Moreover, as some circRNAs can act as coding templates, it is expected that applying their expression cassettes in tumour suppressor proteins could be another approach to assist antitumour efforts.

### 5.3 Immuno‐oncology

Although both the innate and adaptive immune system function in antitumour response, tumours can escape from immune attack by lowing the exposure of their antigens and reducing infiltration and function of effector T cells and dendritic cells (DCs). Despite recent achievements of diverse antitumour immunotherapy, including checkpoint blockades, cellular therapies, cytokines and tumour vaccines, there are still a variety of issues to be resolved, partially due to tumour heterogeneity and side effects.^73^ The emerging functions of circRNAs in tumour development and immune regulation give them great potential in antitumour immunotherapy.

To investigate influence of circRNAs on natural killer (NK) cells, Ma et al^74^ used a specific plasmid for CircARSP91 overexpression and analysed the response of hepatocellular carcinoma (HCC) cells to NK cell cytotoxicity. They identified the up‐regulated UL16 binding protein 1 (ULBP1), which could impact NK cell activation. Ultimately, they concluded that CircARSP91 can strengthen NK cell antitumour cytotoxicity by up‐regulating ULBP1.

As described above, Zhang et al^26^ reported that the overexpression of hsa_circ_0020397 in CRC cells could promote expression of TERT and PD‐L1, which contribute to immunocyte exhaustion and tumour escape from immune responses by sponging miR‐138. This provides us with new insights on “checkpoint therapy” in cancer patients, where alteration of specific circRNA expression may improve the effect of checkpoint therapy.

Due to the distinct properties of stability and specificity, circRNAs in different tumour tissues may act as tumour antigens for immune response. Moreover, circRNAs may carry tumour‐specific miRNAs or mRNAs as novel tumour antigens transported from the tumour cells to the recipient cells through exosomes and extracellular vesicles.^41^, ^60^, ^75^ As coding template, proteins or peptides translated from abnormal circRNAs may also act as tumour antigens.^19^, ^58^ As is known, for cancer vaccine therapy or CAR T cell adoptive therapy, it is more attractive to explore novel and specific antigens to improve the treatment outcome. In this way, this new potential tumour antigen may be applied in cancer vaccine therapy or CAR T cell adoptive therapy.

Because research on miRNAs in antitumour immunity is clearer than that on circRNAs, the close relationship between circRNAs and miRNAs can help research into the role of circRNAs in antitumour immunity. Using circRNA databases (starBase v2.0 and circBasecan), we can predict a potential circRNA regulating tumour immunity‐associated miRNAs and identify potential new tumour antigens.^76^, ^77^ In addition to the circRNA‐miRNA network, some circRNAs indirectly contribute to antitumour immune responses by affecting some proteins. For example, Du et al^12^ found that endogenous circ‐Foxo3 could elicit the degradation of p53, modulating immune response, by formation of a complex with MDM2. Silencing of circ‐Foxo3 could increase cell viability, whereas overexpression of circ‐Foxo3 could induce apoptosis and suppress tumour growth. These circRNAs identified in tumour immunity can be potential targets for antitumour therapy.

Taken together, this evidence exhibits the potential of circRNAs in regulation of antitumour immune responses and even as therapeutic targets. However, the underlying mechanisms of circRNAs in this field remain largely unknown and need to be further clarified.

Additionally, with increased recognition of circRNAs in antitumour‐drug resistance, targeting specific circRNA may ameliorate the drug resistance during antitumour treatment. As mentioned above, up‐regulated circPVT1 in gastric cancer is associated with multidrug resistance, such as resistance to cisplatin, paclitaxel, and oxaliplatin; thus, targeting circPVT1 may have the potential to treat drug resistance in gastric cancer.

## 6 CHALLENGES IN CANCER THERAPY

Using circRNAs as novel therapeutic targets will undoubtedly expand the field of potential “druggable” targets. However, despite the rapid growth of related research in recent years, knowledge of circRNAs is not sufficient compared to that of miRNAs and mRNA. Therefore, prior to clinical application, we should investigate the issues and challenges that currently exist in antitumour therapy.

### 6.1 Potential targets

Simply, discovery of a novel circRNA in one type of cancer cell or tissue and experimentally finding that it acts as an oncogene or tumour suppressor does not rule out the possibility that the circRNA may be involved in other vital physiological processes or has other targets that have yet to be identified. In addition, one circRNA may target multiple miRNAs in one cancer; for example, circ‐ITCH can sponge both miR‐7 and miR‐214 to inhibit the Wnt/β‐catenin pathway and repress proliferation of lung cancer cells.^28^ Additionally, a specific circRNA may be down‐regulated in different tumours, which implies that its dysregulation cannot be uniquely related to a specific disease.^12^, ^68^ Furthermore, the same circRNA may exert opposite functions by targeting different miRNAs in different cancers. CircHIPK3 can target miR‐558 to down‐regulate HPSE and subsequently inhibit angiogenesis, migration and invasion of BC cells, but also can target miR‐7 to promote CRC growth and metastasis.^78^, ^79^ Thus, it is important to study the circRNA spectra as much as possible before focusing on single molecules related to a certain disease and to carry out enough trials to minimize the harm induced by other potential targets.

### 6.2 Specificity

Specificity is one of the most important matters to consider when drugs or therapeutic targets are applied clinically so as to avoid possible off‐target effects and assure safety and efficacy. First, targeting tumour‐associated circRNAs should preferably be exerted without disturbing expression of other RNAs, especially the corresponding linear mRNA. RNAi and ASOs are currently the two main approaches used to target RNAs, and minimizing the toxicity of off‐target is a major concern.^80^ Considering the structure and biogenesis of circRNAs, RNAi or ASOs should be designed to be perfectly complementary to either the unique back‐splice junction site or the back‐splice signals in the pre‐mRNA, such as binding sites for trans‐acting splicing factors or flanking intronic Alu repeats.^4^ Second, as mentioned above, circRNA has tissue‐, cell‐ and developmental stage‐specific expression patterns, which should be taken into account when targeting circRNAs in antitumour therapy. Recently, Lasse et al expanded the knowledge about spatially resolved cellular expression patterns of ciRS‐7 in colon cancer.^81^ They were the first to identify not only the complete absence of ciRS‐7 in cancer cells, but also high expression in stromal cells within the tumour microenvironment, which provides guidance when selecting a target cell when targeting ciRS‐7 to treat colon cancer.

### 6.3 Gene therapy

In gene therapy, overexpression of circRNA is common, and plasmid or lentiviral vectors are frequently used.^82^ During circRNA formation, inverted repeats, especially Alu repeats, are one of the most important factors to support successful circularization.^4^ Thus, the cloned sequences contain the interesting exon(s) and splice sites flanked either by cognate Alu repeats or artificial long inverted repeats. The key point is to avoid the introduction of foreign circRNA, such as synthetic or exogenous circRNAs, which can stimulate RIG‐I signalling as nonself‐molecules and elicit immune responses. Unlike the endogenous circRNAs, exogenous circRNAs possess few RNA associated proteins. Moreover, the intracellular delivery of exogenous circRNA may induce a fast up‐regulation of various inflammation‐associated genes. In this sense, the mechanism for distinguishing between self and nonself circular RNAs should be explored by further research.^57^ Alternatively, finding a proper delivery system may be taken into consideration as an optional strategy.

### 6.4 Delivery

How to safely deliver engineered circRNAs in vivo is an important question. As mentioned above, the abundance and stability of circRNAs in exosomes has been identified. Exosomes have a lipid bilayer structure, which can protect RNAs against degradation and ensure their effective concentration.^8^ Furthermore, the small size and membrane structure of exosomes facilitate their absorption and fusion by cancer cells. Transporting circRNAs via exosomes can support intercellular communication in cancer.^19^ For therapy purposes, modified exosomes may be an effective approach, because they contain engineered circRNAs or siRNAs targeting circRNA.^83^ However, even if cancer‐derived exosomes have higher affinity with malignant cells, the possibility of targeting healthy ones still cannot be excluded. In recent times, nanoparticles, as a promising tool to deliver nucleic acids into cells, have emerged for application in therapy. Du et al^12^ reported the application of gold nanoparticles to deliver circ‐Foxo3 in mice to inhibit tumour progression. However, this approach can only be applied to exonic circular RNAs, because nanoparticles have no ability to enter the nucleus. A recent study of combination of nanoparticles and microfluidics may provide an exciting strategy to increase nuclear entry for resolving this limitation.^84^ Hence, it is expected that circular RNAs will become a new therapy target or tool to treat diverse cancers as the field continues to grow and delivery problems are resolved.

### 6.5 Current progress

Although various studies have indicated the promising potential of circRNAs in cancer therapy (Table 1), such as in gene therapy, immunotherapy and a combination of the two, there are still many drawbacks to overcome. Thus far, there have been no preclinical reports in which circRNAs are applied alone as therapeutic targets or vectors in cancer, but we believe the ongoing research will change this in the future. With the development of new technologies, more circRNAs will be discovered as diagnosis‐ and prognosis‐related markers, and these can be used to guide clinical medication selection. Therapeutic strategies that depend on circRNAs will be formulated, thereby providing new perspectives and directions for cancer therapy. Each experimental report will bring us closer to a patient‐tailored approach to achieve maximum anticancer effects with minimal side effects. Additional work with larger sample sets and long‐term follow‐up clinical information is needed for further validation.

**Table 1:** Current Circular RNA in cancer therapy

Tumour type	CircRNA	Function	Mechanism/Experiments	Ref.
*Down‐regulation*				
Hepatocellular carcinoma	circARSP91	Interaction with protein	circARSP91/UL16 binding protein1. Overexpression of circARSP91 could enhance the cytotoxicity of natural killer cells against hepatocellular carcinoma	74
	cSMARCA5	MiRNA sponge	cSMARCA5/ miR‐17‐3p and miR‐181‐5p/ TIMP metallopeptidase inhibitor 3. Overexpression of cSMARCA5 suppresses the proliferation and migration of HCC cells	33
	hsa_circ_0079299	Interaction with protein	Inhibiting cell proliferation through PI3K/AKT/mTOR signalling pathway. Over‐Overexpression of hsa_circ_0079299 suppressed tumour growth in vitro and in vivo, retarded cell cycle progression while had no effect on cell migration and apoptosis	85
Bladder cancer	circ_ZKSCAN1	MiRNA sponge	circ‐ZKSCAN1/miR‐1178‐3p/p21. Overexpressed circ‐ZKSCAN1 inhibited cell proliferation, migration, invasion and metastasis in vitro and in vivo	86
	circHIPK3(BCRC‐2)	MiRNA sponge	circHIPK3/ miR‐558/heparinase. Overexpression of circHIPK3 effectively inhibited migration, invasion, and angiogenesis of bladder cancer cells in vitro and suppresses bladder cancer growth and metastasis in vivo.	78
	circ‐Foxo3	MiRNA sponge	circ‐Foxo3/miR‐191‐5p. Overexpression of circ‐Foxo3 promoted bladder cancer cell apoptosis in BBN mice and in human bladder cancer cell lines	68
Breast cancer	circ‐Foxo3	Interaction with protein	the complex of circ‐Foxo3 and MDM2 induces the degradation of p53, modulating immune responses during tumorigenesis. Ectopic expression of circ‐Foxo3 triggered stress‐induced apoptosis and inhibited the growth of tumour xenograft.	12
Gastric cancer	circ_100269	MiRNA sponge	CircRNA_100269/miR‐630/LPHN2. Overexpression suppresses tumour cell growth.	87
	hsa_circ_0000096	Interaction with protein	modulating cyclin D1, CDK6, matrix metalloproteinase 2 (MMP‐2), and MMP‐9. Knockdown of hsa_circ_0000096 significantly inhibited cell proliferation and migration in vitro and in vivo	88
	circFAT1(e2)	MiRNA sponge in cytoplasm and interaction with protein in nucleus	circFAT1(e2)/miR‐548g/RUNX1 in the cytoplasm and targeting YBX1 in the nucleus. Overexpression of circFAT1(e2) inhibiting proliferation, migration and invasion of gastric cancer cells	31
	circMRPS35	Interaction with protein even directly mRNA regulator	circMRPS35/KAT7/FOXO1/3a. recruit KAT7 to and directly bind to FOXO1/3a promoter region. circMRPS35 overexpression suppressed the proliferation and invasion of gastric cancer cells in vitro and in vivo	89
Kidney cancer cell	circC3P1	MiRNA sponge	circC3P1/ miR‐21/PTEN and inactivating PI3K/AKT and NF‐κB signalling pathways. CircC3P1 overexpression declined cell viability, migration, and invasion and caused apoptosis	90
Lung cancer	circPTK2	MiRNA sponge	circPTK2/miR‐429, miR‐200b‐3p/TIF1γ.CircPTK2 overexpression inhibited TGFβ‐induced EMT	91
	circNOL10	Interaction with protein	circNOL10 inhibits lung cancer development by promoting SCLM1‐mediated transcriptional regulation of the humanin polypeptide family in vitro and in vivo	92
	circRNA‐ITCH	MiRNA sponge	circRNA‐ITCH/ miR‐7, miR‐17 and miR‐214/ ITCH, and suppress the activation of Wnt/‐catenin signalling. Overexpressed circRNA‐ITCH suppress lung cancer cell proliferation	28
Colorectal cancer	hsa_circ_0014717	MiRNA sponge	hsa_circ_0014717/p16. Hsa_circ_0014717 overexpression could significantly suppress CRC cell proliferation and colony formation, as well as induce cell cycle G0/G1 phase arrest in vitro and inhibit xenograft tumour growth in vivo	93
Glioblastoma	circ‐FBXW7	Translation	Translation template of FBXW7‐185aa. Up‐regulation of FBXW7‐185aa can help to inhibit proliferation and cell cycle acceleration	16
Prostate cancer	circAMOTL1L	MiRNA sponge	circAMOTL1L/miR‐193a‐5p/Pcdha.CircAMOTL1L overexpression inhibited progression and invasion of prostate cancer cells	32
Rectal cancer	circMTO1	MiRNA sponge	circMTO1/miR‐19b‐3p/JAK1/STAT3 and AMPK signal pathways. CircMTO1 overexpression could suppress cell proliferation, migration, and incursion and induce apoptosis	94
	CDR1as (or CiRS‐7)	MiRNA sponge	CDR1as/miR‐1270/SCAI signalling pathway. Overexpression of CDR1as sensitized ovarian cancer to cisplatin, inhibited cell proliferation and promoted the cisplatin‐induced cell apoptosis in ovarian cancer cells	95
	CDR1as (or CiRS‐7)	MiRNA sponge	CDR1as/miR‐135b‐5p/HIF1AN.CDR1as overexpression inhibited the proliferation, invasion and migration of ovarian cancer cells	96
*Up‐regulation*				
Pancreatic cancer	hsa_circ_0020397	MiRNA sponge	hsa_circ_0020397/miR138/TERT and PD‐L1, contributes to immunocytes exhaustion and tumour escape from immune responses, new insight for "checkpoint therapy"	26
Breast cancer	hsa_circRNA_002178	MiRNA sponge	hsa_circRNA_002178/miR‐328‐3p/COL1A1.Hsa_circRNA_002178 silencing inhibited inflammation in vivo through reducing TNF‐α and IL‐6 levels and prevented tumour growth	53
	circ‐AMOTL1	Interaction with protein	Facilitate c‐myc nuclear translocation and regulate its gene transcription. Silencing circ‐Amotl1 decreased cancer cell proliferation but increased cell apoptosis	70
Cervical cancer	circAMOTL1	MiRNA sponge	circAMOTL1/miR‐485‐5p/AMOTL1. Gain‐ or loss‐of‐function assays and in vivo experiments demonstrated that AMOTL1 promoted cervical cancer cell growth both in vitro and in vivo	97
Colorectal cancer	circPTK2	Interaction with protein	circPTK2 binding to protein vimentin. Silencing circPTK2 blunt tumour metastasis in a patient‐derived CRC xenograft model	98
	circHIPK3	MiRNA sponge	circHIPK3/miR‐7/c‐Myb. Knockdown of circHIPK3 markedly inhibited CRC cells proliferation, migration, invasion, and induced apoptosis in vitro and suppressed CRC growth and metastasis in vivo	79
Colorectal cancer, osteosarcoma, hepatocellular carcinoma	CDR1as (or CiRS‐7)	MiRNA sponge	CDR1as (or CiRS‐7)/ miR‐7/EGFR, CCNE1, PI3KCD, and RAF1, promotion of tumorigenesis and invasion. CDR1as inhibition in vivo also induced tumour regression	99
Non‐small‐cell lung carcinoma	CDR1as (or CiRS‐7)	MiRNA sponge	CDR1as/miR‐219a‐5p/SOX5. Knockdown of circCDR1as inhibited the progression of NSCLC by decreasing cell viability, migration and invasion and increasing apoptosis	100
Gastric cancer	CDR1as (or CiRS‐7)	MiRNA sponge	CDR1as/miR‐7‐5p/REGγ. Knock‐down of CircRNA CDR1as specifically promoted the cytotoxic effects of low‐dose DB on GC cells instead of hepatocytes.	101
	hsa_circ_0000291	MiRNA sponge	hsa_circ_0000291/miR‐183/ITGB1. Silencing hsa_circ_0000291 suppressed GC cell metastasis and proliferation both in vivo and in vitro	69
	circ‐RanGAP1	MiRNA sponge	circ‐RanGAP1/miR‐877‐3P/VEGFA. Inhibition of circ‐RanGAP1 decreased GC cell invasion and migration in vitro	35
	circ_PVT1	MiRNA sponge	circ‐PVT1/miR‐124‐3p/ ZEB1. Circ‐PVT1 knockdown increased PTX sensitivity of GC in vivo	102
	circ_PVT1	MiRNA sponge	circPVT1/miR‐199a‐5p/YAP1 and PI3K/AKT pathways. Silencing hsa_circ_PVT1 (circPVT1) suppressed the growth and metastasis of glioblastoma multiforme cells	103
Osteosarcoma	circ_PVT1		Knockdown of circPVT1 can weaken the resistance to doxorubicin and cisplatin of OS cells through decreasing the expression of ABCB1	61
Gliomas	circ‐TTBK2	MiRNA sponge	circ‐TTBK2/miR‐217/ HNF1β/Derlin‐1. Knockdown of circ‐TTBK2 combined with miR‐217 overexpression can suppress tumorigenesis in vivo	104

Note

Reported circRNAs that have been identified to have tumour therapeutic potential in in vitro or in vivo experiments via different strategies, mainly gene therapy and immunotherapy.

## CONFLICT OF INTEREST

The authors confirm that there are no conflicts of interest.

## AUTHOR CONTRIBUTION

**Weizhen Li:** Writing‐original draft (lead); Writing‐review & editing (lead). **Jia‐Qiang Liu:** Formal analysis (equal); Project administration (equal); Resources (supporting); Writing‐review & editing (equal). **Ming Chen:** Funding acquisition (equal); Project administration (equal); Resources (equal). **Jiang Xu:** Funding acquisition (equal); Project administration (equal); Resources (equal); Writing‐review & editing (equal). **Di Zhu:** Conceptualization (lead); Formal analysis (lead); Funding acquisition (equal); Project administration (lead); Resources (equal); Supervision (equal); Writing‐original draft (lead); Writing‐review & editing (lead).

## Data Availability

The datasets generated during and/or analysed during the current study are available in the figshare repository.
